# CAR-T cell therapy based on a TCR mimic nanobody targeting an intracellular antigen for solid cancers

**DOI:** 10.1016/j.omton.2025.200948

**Published:** 2025-02-18

**Authors:** Qingqing Lin, Shi Jin, Mingjun Wang

**Affiliations:** 1 Department of Research and Development, Shenzhen Institute for Innovation and Translational Medicine, Shenzhen International Biological Valley-Life Science Industrial Park, Dapeng New District, Shenzhen, China; 2 Shenzhen Innovation Immunotechnology Co., Ltd., Shenzhen International Biological Valley-Life Science Industrial Park, Dapeng New District, Shenzhen, China; 3 National Cancer Center/National Clinical Research Center for Cancer/Cancer Hospital & Shenzhen Hospital, Chinese Academy of Medical Sciences and Peking Union Medical College, Shenzhen 518116, China; 4 Department of Medical Oncology, South China Hospital of Shenzhen University, Shenzhen, China

## Main text

Conventional chimeric antigen receptor (CAR)-T cell therapy has shown remarkable success in treating hematological malignancies, such as acute lymphoblastic leukemia and lymphoma, but it has poor efficacy in solid cancers due to the lack of ideal surface antigens.^1^ Duan et al. evaluated two novel T cell receptor mimic (TCRm) nanobodies (F5 and G9) targeting the HPV16 E6_29–38_ peptide complexed with human leukocyte antigen (HLA)-A∗02:01 in the CAR format.^2^ CAR-T cells based on the F5 nanobody specifically killed target cells *in vitro* and inhibited the tumor growth *in vivo*, suggesting that CAR-T cells based on TCRm might provide a strategy not only for the treatment of HPV16+ malignancies but also for other types of solid cancers.

As of January 2025, there are 7 CAR-T cell products approved in the USA and 6 CAR-T cell products approved in China. Since conventional CAR-T cells recognize tumor cells through interactions between an artificial single-chain variable fragment (scFv) and surface tumor antigens, when CAR-T cell therapy is applied to treat patients with solid cancers, very limited therapeutic efficacy could be observed, and the lack of appropriate surface targets is one of the main reasons.^1^ TCR-T cell therapy functions through TCR-peptide-major histocompatibility complex (pMHC) interactions and has achieved promising clinical efficacy in multiple solid tumors.^3^ Peptides derived from either surface antigens or intracellular proteins can be presented by MHC molecules for recognition by TCR-T cells; therefore, TCR-T cells can recognize a wider range of antigens as targets and provide great promise in treating solid tumors. The first TCR-T cell product was approved recently in August 2024. In addition, in 2022, a TCR-based drug called KIMMTRAK was approved by the FDA for treating unresectable or metastatic uveal melanoma. Evidently, the construction of CAR-T cells based on TCRm antibodies targeting an intracellular protein-derived peptide complexed with HLAs is a strategy for enhancing the efficacy of CAR-T cell therapy in treating solid cancers.

**Figure 1:**
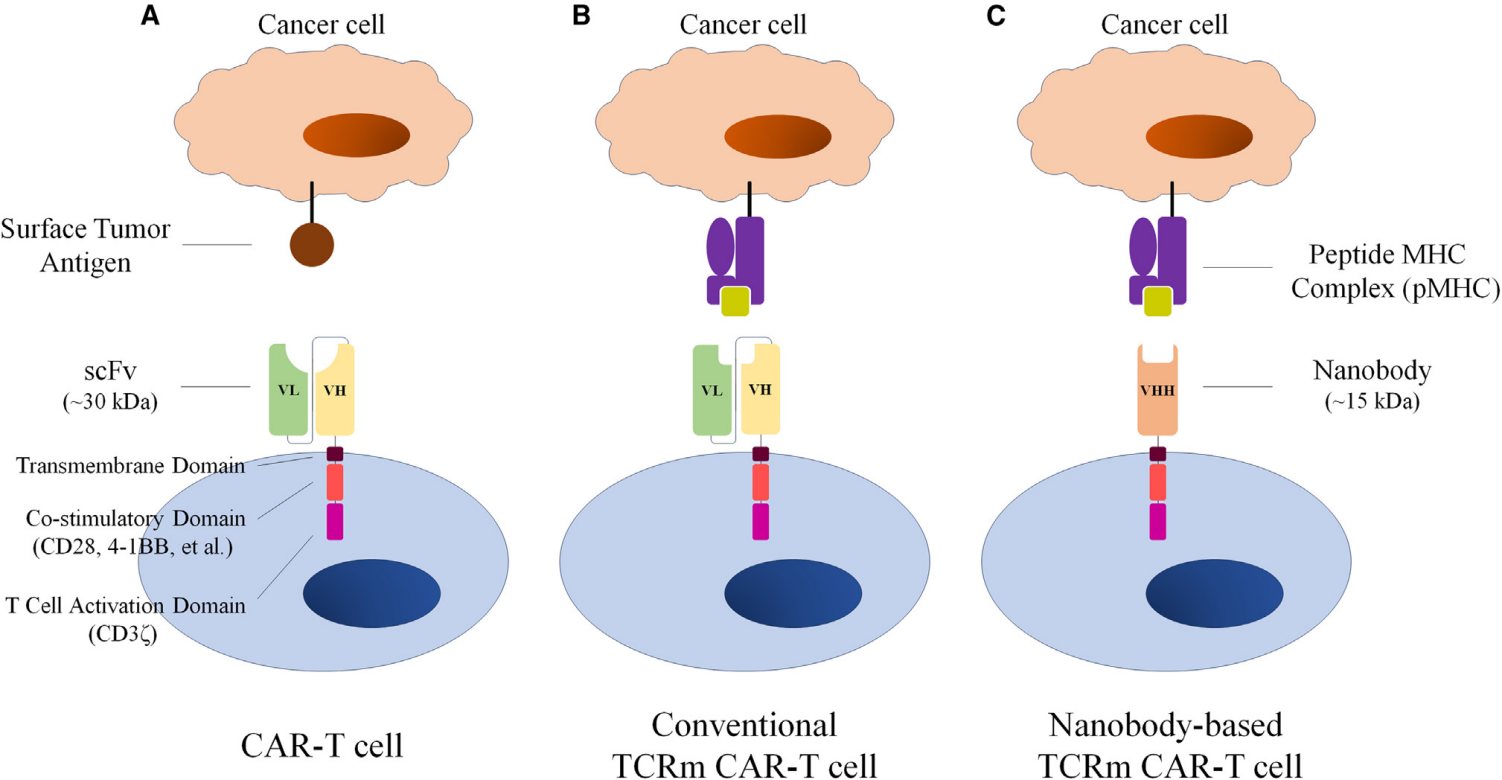
Construction of various types of CAR-T cell designs CAR-T cells recognize tumor cells through an artificial single-chain variable fragment (scFv), a fusion protein of VH and VL from immunoglobulins (A). Conventional TCRm-based CAR-T cells use scFv to recognize intracellular protein by the relevant peptide MHC complex (B). TCRm nanobody-based CAR-T cells utilize the nanobody, which only contains a variable domain of heavy chain (V_H_H) and has an easier time targeting the buried epitope in pMHC due to its smaller size (around 15 kDa), to target pMHC on cancer cells (C).

Since a TCRm antibody targeting a human tumor antigen was constructed in 2000, TCRm antibodies have been reported in a number of studies,^3^^,^^4^^,^^5^ and CAR-T cells based on TCRm targeting NY-ESO-1/HLA-A2 were described as well.^6^ A proper tumor antigen should be highly expressed in tumor cells with low or no expression in normal tissues. E6 and E7 proteins of HPV16 are ideal targets for immunotherapy: (1) they are expressed in cancer cells but not normal cells, (2) they are foreign antigens with high immunogenicity, and (3) they are uniformly and constitutively expressed in cancer cells. Although a TCRm antibody directed against HPV16 E7 was reported in 2022,^7^ the TCRm antibody targeting HPV16 E6 had not been reported previously. TCRm antibodies were conventionally produced from a human antigen-binding fragment (Fab) or scFv phage-displayed library.^6^ However, these conventional antibodies could not efficiently reach the epitopes buried in the antigen-binding groove of MHC; therefore, Duan et al. chose nanobodies to solve the problem.^2^ A nanobody only contains variable domain of heavy chain (V_H_H) (Figure 1) and can specially target buried epitopes due to their small size (around 15 kDa). In addition, caplacizumab, a humanized nanobody targeting von Willebrand factor (vWF),^8^ was approved for the treatment of acquired thrombotic thrombocytopenic purpura (aTTP) in 2019 by the FDA, indicating the clinical safety and efficacy of the nanobody. However, the safety and efficacy of the TCRm nanobody-based CAR-T cells, which were evaluated in the murine model by Duan et al.^2^, remain to be tested in human patients.

Although CAR-T cells based on TCRm antibodies have advantages over traditional CAR-T cells in terms of treatment for solid cancers, some caveats remain to be addressed. Firstly, just like TCR-T cell therapy, TCRm antibodies still face the challenges of MHC restriction and the downregulation of HLA molecules in tumor cells. Secondly, although TCRm antibodies can greatly broaden the tumor antigen candidates for CAR-T cell therapy against solid cancers, good tumor antigen targets are still limited, which is a barrier to cancer immunotherapies. Thirdly, although camel nanobodies can be humanized to reduce their immunogenicity, the rejection of camel nanobodies by the human immune system needs to be carefully evaluated.

In summary, Duan et al. demonstrated that CAR-T cells based on a TCRm nanobody targeting the HPV16 E6_29–38_ peptide complexed with HLA-A∗02:01 can kill the cervical cancer cell lines *in vitro*, *in vivo*, and *ex vivo*, providing an approach for the treatment of HPV16+ malignancies.^2^ In addition, this study suggests that CAR-T cells based on TCRm nanobody targeting the other tumor antigen-derived peptides complexed with different HLA molecules might be a promising strategy for treating various types of solid cancers as well.

## Acknowledgments

M.W. received supports from Shenzhen Major Science and Technology Special Project, China (KJZD20240903102713018) and the Shenzhen Science and Technology Research and Development Fund, China (CJGJZD20200617102403009). S.J. received supports from the Shenzhen Science and Technology Program, China (RCJC20200714114436049) and the Cancer Hospital, 10.13039/501100005150Chinese Academy of Medical Sciences, Shenzhen Center/Shenzhen Cancer Hospital Research Project, China (SZ2020ZD006).

## Author contributions

M.W., S.J., and Q.L. conceived and wrote this commentary.

## Declaration of interests

The authors declare no competing interests.
